# Explainable machine learning for orthopedic decision-making: predicting functional outcomes of total hip replacement from gait biomechanics

**DOI:** 10.1186/s13075-025-03709-2

**Published:** 2025-12-23

**Authors:** Bernd J. Stetter, Jonas Dully, Felix Stief, Jana Holder, Hannah Steingrebe, Frank Zaucke, Stefan Sell, Stefan van Drongelen, Thorsten Stein

**Affiliations:** 1 Institute of Sports and Sports Science, Karlsruhe Institute of Technology, Karlsruhe, Germany; 2 Department of Sports Science, University of Kaiserslautern-Landau (RPTU), Kaiserslautern, Germany; 3 Function and Motion Lab, Berufsgenossenschaftliche Unfallklinik Frankfurt, Frankfurt am Main, Germany; 4 Department of Sport and Exercise Science, University of Salzburg, Hallein, Salzburg, Austria; 5 Dr. Rolf M. Schwiete Research Unit for Osteoarthritis, Department of Trauma Surgery and Orthopedics, University Hospital Frankfurt, Goethe University Frankfurt, Frankfurt am Main, Germany; 6 Joint Center Black Forest, Hospital Neuenbuerg, Neuenbuerg, Germany

## Abstract

This study aimed to identify subpopulations of patients with hip osteoarthritis who exhibit distinct adaptations in gait biomechanics, and to evaluate subpopulation-specific effects of total hip replacement on gait biomechanics. Three datasets were analyzed: (1) a cohort of 109 unilateral hip osteoarthritis patients before total hip replacement, (2) a subset of the first dataset of 63 patients re-evaluated after total hip replacement and (3) a control group of 56 healthy individuals. For all participants, three-dimensional joint angle and moment waveforms of the pelvis, ipsilateral hip and knee, as well as sagittal-plane ankle motion and the foot progression angle, were obtained. The analytical framework integrated k-means clustering, support vector machine classifiers, Shapley Additive exPlanations, and statistical waveform analyses. Clustering of the pre-operative dataset revealed three distinct subpopulations characterized by unique patterns in gait kinematics and joint moments. These subpopulations also differed in age, Kellgren-Lawrence score, and walking speed. Prior to total hip replacement, between 51.4% and 85.2% of hip osteoarthritis patients were classified as pathologic; following surgery, this proportion decreased to 27.8% − 51.8%. Hip flexion and rotation angles and moments were identified as the most important features for patient classification. The magnitude of gait improvement after total hip replacement varied across subpopulations, indicating subpopulation-specific responses to surgical intervention. In conclusion, patients with hip osteoarthritis demonstrate distinct subpopulation-specific gait adaptations, both before and after total hip replacement. Preoperative classification of patients into the identified subpopulations using machine learning approaches may facilitate the prediction of postoperative gait recovery and support the development of personalized treatment and rehabilitation strategies.

**Supplementary Information:**

The online version contains supplementary material available at 10.1186/s13075-025-03709-2.

## Introduction

Hip osteoarthritis (OA) is a degenerative joint disease and a major public health concern [1]. Its prevalence is expected to increase due to demographic aging, as well as rising obesity and injury rates [2]. Hip OA is a progressive condition [3] and as it progresses, patients typically experience a decline in quality of life [4]. When quality of life becomes severely affected, total hip replacement is the primary surgical treatment option [5]. Recent advances in machine learning (ML) have created new opportunities to enhance clinical decision-making in OA and joint replacement [6, 7]. For instance, ML models haven been used to predict patient dissatisfaction following total knee replacement by combining clinical and imaging data to identify individuals less likely to benefit from surgery [6].

Biomechanical alterations in gait kinematics and kinetics are well documented in patients with hip OA [8, 9]. Many patients exhibit compensatory changes in hip kinematics [8], while reductions in joint moments are particularly characteristic for patients with end-stage hip OA [9]. Although gait biomechanics generally improve within one year after total hip replacement [10], some limitations - such as decreased stride length and diminished hip range of motion in the sagittal plane - often persist [10, 11]. Walking speed, an important indicator of functional capacity [12], typically recovers after surgery [13]. However, postoperative adaptations are influenced by preoperative gait patterns [14], meaning that certain biomechanical effects may remain undetected when using conventional analyses. Furthermore, most existing studies have focused on discrete biomechanical parameters (e.g. peak values or range of motion) rather than continuous waveform data. Because these gait waveforms contain hundreds of data points, they provide a more comprehensive representation of movement patterns [15].

The high dimensionality of biomechanical waveforms can be effectively addressed using ML-based analysis techniques [16]. Machine learning enables the identification of subgroups within complex datasets that are internally similar but distinct to other groups, a process known as clustering [17]. Previous studies used ML to identify patient subpopulations exhibiting similar gait compensating strategies [14] and to evaluate treatment effects [18]. In a prior study from us, clustering based on lower-body kinematics identified two characteristic subpopulations among patients with hip OA, offering a more nuanced understanding of movement biomechanics [14]. Extending this approach to a larger and more diverse dataset, including variables like joint moments, may uncover additional clinically meaningful subpopulations. Identifying and characterizing these biomechanical subpopulations could support optimized treatment decisions and more personalized rehabilitation strategies following total hip replacement [10, 14]. Machine learning can facilitate this biomechanical characterization, particularly because deviations from healthy gait can be subtle [19]. Support vector machines (SVM), for example, have demonstrated high sensitivity in detecting systematic changes in gait biomechanics [20, 21]. Furthermore, novel ML-derived metrics such as the classifier-oriented gait score (COGS) [18] provide low-dimensional, clinically interpretable measures designed to support practical clinical applications through assistive tools. Such ML-based tools can automatically evaluate complex biomechanical gait data, providing clinicians with a more streamlined, data-driven assessment of gait function [22]. Despite growing integration of ML into biomechanical research [15, 16], its clinical application remains limited due to the black-box nature of many ML techniques [23]. Consequently, explainable ML [24], focused on building interpretable, white-box models, has gained increased attention. Explainable ML has recently been applied to understand biomechanical differences between patients after total hip replacement and healthy participants from classification models [25]. However, the combined use of data-driven subpopulation identification based on gait biomechanics and the subpopulation-specific assessment of treatment effects using explainable ML has not yet been explored.

Therefore, the present study aimed to identify and characterize subpopulations of patients who respond differently to hip OA by exhibiting distinct adaptations in gait kinematics and joint moments using clustering techniques, SVMs and explainable ML. It was hypothesized that (1) the identified subpopulations would differ in their patient characteristics and gait biomechanics relative to healthy controls, and (2) total hip replacement would lead to subpopulation-specific changes in patient characteristics, gait biomechanics, and COGS.

## Methods

### Participants

The analysis included three datasets drawn from our current and previous prospective studies [26–29]: one comprising 109 unilateral hip osteoarthritis patients before total hip replacement (HOA), a subset of 63 patients who were re-examined 7–25 months after total hip replacement (THR), and a dataset of 56 healthy controls (HC). The protocols for the original studies were approved by local Medical Ethics Committee (reference number 122/14, 497/15 and 2021-52). A detailed description of the three datasets is presented in Table 1.

**Table 1:** Descriptive data of the three investigated datasets

	HOA (*N* = 109)	THR (*N* = 63)	HC (*N* = 56)
Sex (male/female)	56/53	34/29	23/33
Age [years]	61.3 ± 10.7	62.4 ± 9.9	63.5 ± 7.6
Body mass [kg]	80.5 ± 14.6	82.3 ± 13.7	69.0 ± 11.9
Body height [m]	1.70 ± 0.09	1.72 ± 0.07	1.68 ± 0.09
BMI [kg/m^2^]	27.7 ± 4.1	28.1 ± 4.0	24.2 ± 2.8
Affected leg (Left/Right)	46/63	30/33	26/30
Walking speed [m/s]	1.01 ± 0.19	1.17 ± 0.15	1.32 ± 0.15

For the HC dataset, the variable affected leg represents the total number of left and right legs considered

*HOA *Hip Osteoarthritis dataset, *THR *Total Hip Replacement dataset,* HC *Healthy Controls dataset,* BMI *Body Mass Index

### Data acquisition

Biomechanical data were collected using a 3D motion capturing system (8 MX T10 cameras, 200 Hz; Vicon Motion Systems Ltd., Oxford, United Kingdom) and two AMTI force plates (1000 Hz; Advanced Mechanical Technology, Inc., Watertown, MA, USA). A modified Plug-in-Gait marker set was applied, with additional markers placed on the medial malleolus, medial femoral condyle and trochanter major [30]. Marker trajectories were reconstructed and smoothed using a Woltring filter (mean square error = 10) within the Vicon Nexus software (version 2.12; Vicon Motion Systems, Oxford UK). Kinematic and kinetic data were time-normalized to 101 datapoints for the stance phase of the gait cycle. Joint moments were normalized to body mass and expressed as external moments. For each participant, three barefoot gait trials at self-selected walking speed were analyzed. This represented the maximum number of valid trials available across all participants, thereby ensuring consistency and comparability in the data [31, 32]. For the study groups HOA and THR the affected leg was analyzed. To maintain side-to-side balance between groups, the ratio of left to right affected legs in the HOA group was used to randomly assign the right or left leg for analysis in the HC group.

### Data processing

**Fig. 1:**
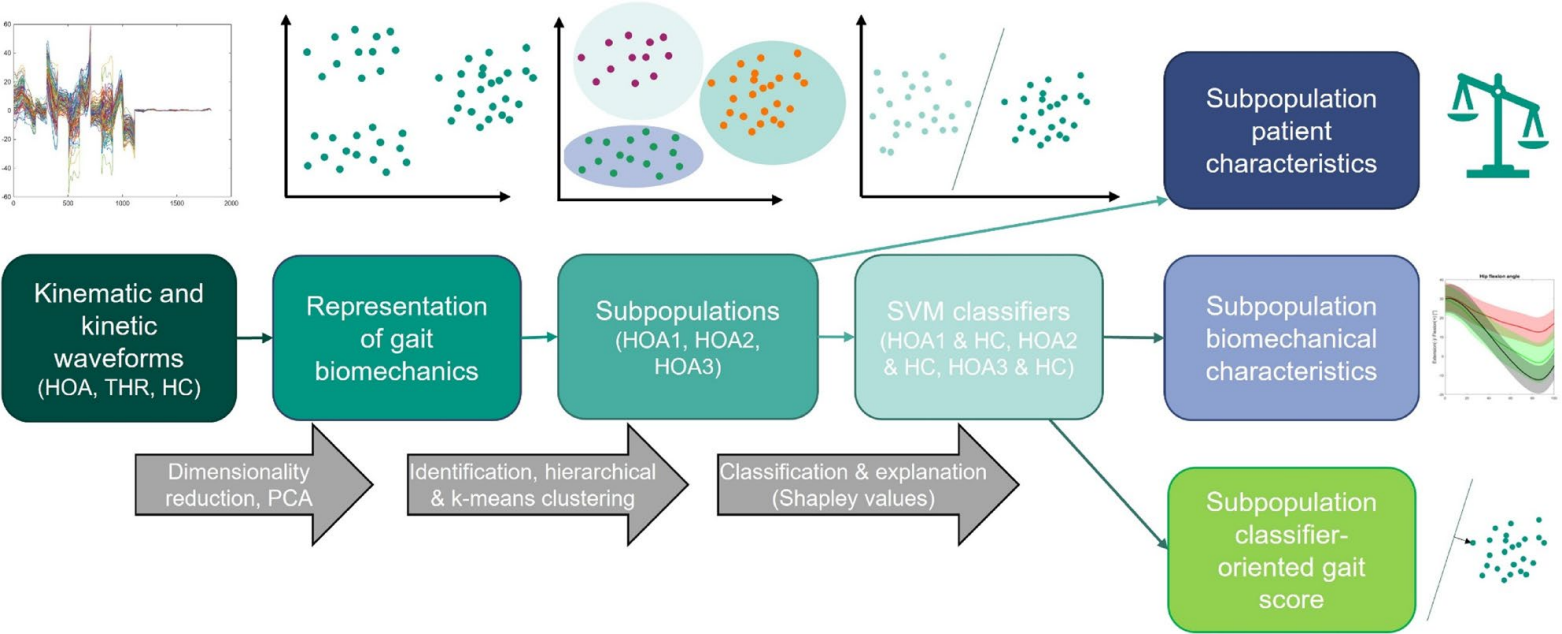
Illustration of the machine learning workflow used to identify and characterize subpopulations of patients with hip osteoarthritis and to assess the subpopulation-specific effects of total hip replacement. Abbreviations: HOA = Hip Osteoarthritis dataset; THR = Total Hip Replacement dataset; HC = Healthy Controls dataset; PCA = Principal Component Analysis; SVM = Support Vector Machine

A visual overview of the study workflow is provided in Figure 1. For each participant, 18 biomechanical waveforms were averaged over the three trials, and the resulting mean waveforms were used for subsequent analyses. Kinematic (joint-angle) waveforms included pelvic tilt, pelvic rotation, pelvic obliquity, hip flexion, hip adduction, hip rotation, knee flexion, knee adduction, knee rotation, ankle plantarflexion, and foot progression angles. Kinetic (joint-moment) waveforms included hip flexion, hip adduction, hip rotation, knee flexion, knee adduction, knee rotation, and ankle plantarflexion moments. Because ML-models operate on absolute numerical values, z-standardization was performed [33]. Specifically, the mean and standard deviation of the HC group were used to standardize the HOA and THR datasets, ensuring that all data were expressed within the same dimensional space as the HC group. Principal component analysis (PCA) was then applied to the HOA dataset, represented as a data matrix of 109 participants, each described by 18 standardized waveforms consisting of 101 time points (18 × 101 = 1818 variables). In accordance with established approaches, the PCA retention threshold was set at 90% cumulative variance to achieve substantial dimensionality reduction while retaining relevant biomechanical information [34, 35]. Applied to the HOA dataset, this criterion resulted in 14 retained principal components (PCs) collectively explaining 90% of the total variance [14, 18]. The resulting PCs provide a lower dimensional representation of the individual gait biomechanics. The applied PCA on the HOA dataset yielded eigenvectors defining a reduced feature space. The THR and HC datasets were subsequently expressed into the same feature space by multiplying their respective data matrices with the eigenvector matrix derived from the HOA dataset. This procedure ensured that all groups were represented within the identical feature space, thereby allowing direct comparison and consistent use of components in subsequent modeling.

### Identification of subpopulations with hip OA

Hierarchical clustering combined with k-means clustering was applied to the HOA dataset, following an established approach for identifying subpopulations in biomechanical data [14, 36]. The Ward method was used within hierarchical clustering to determine the optimal number of clusters [16]. Based on this, k-means clustering was performed to identify patients in the different subpopulations. To ensure robust results despite possible variations from repeated runs, the k-means algorithm was executed five times. The silhouette coefficients, which measure how well each patient fits within their assigned cluster, were evaluated for these clusters [37].

### Biomechanical characterization of subpopulations

For each HOA subpopulation, a SVM with a linear function was trained to discriminate between the specific subpopulation and the HC, using a 10-fold-cross-validation. To prioritize model explainability, the box constraint and kernel scale were both set to 1, following the approach used in the COGS study [18]. Classification rates were defined as the percentage of individuals correctly classified. The pathologic ratio was computed as the proportion of individuals within each subpopulation classified as having HOA or THR relative to the total number of individuals in that subpopulation. To further assess model performance, sensitivity (true-positive rate; proportion of pathological cases correctly identified), specificity (true-negative rate; proportion of healthy controls correctly identified), and ROC-AUC (a unitless measure of discriminative ability across all decision thresholds) were calculated. The THR data from each subpopulation were then projected into the corresponding SVM model and evaluated to quantify the postoperative changes following total hip replacement.

Because PCs represent abstract features of the original biomechanical waveforms, direct biomechanical interpretation of differences between the subpopulations and HC is not possible. Therefore, Shapley Additive exPlanations [38] were computed for each PC to identify the most important biomechanical features contributing to the SVM classifiers. The absolute Shapley value of each PC was multiplied by the corresponding PC score, and these weighted PCs were then multiplied by the eigenvector matrix to reconstruct the original biomechanical waveforms. The reconstructed waveforms were subsequently scaled by their importance factor and the mean of these reconstructed waveforms was used to quantify the importance of each original biomechanical waveform. These importance values were model-specific and thus not directly comparable across subpopulations. A higher importance value indicated greater contribution of a given biomechanical waveform in distinguishing the subpopulation from the HC group. For each subpopulation, the five most influential waveforms (i.e., those with the highest importance values) were analyzed to biomechanically characterize them.

The COGS was computed to quantify the gait quality in a low-dimensional space. This score was defined as the orthogonal (minimal) distance of each participant to the SVM hyperplane. More detailed descriptions and visualization are provided in Christian et al. [18].

### Statistical analysis

All statistical analyses were performed in R (version 4.4.1, R Foundation for Statistical Computing, Vienna, Austria) [39]. Normality of all continuous variables was tested using the Shapiro-Wilk test and visual inspection of the Q-Q-plots, while homoscedasticity was evaluated using the Levene test.

Patient characteristics were compared both between subpopulations and HC, and between HOA and THR. Two separate one-way ANOVAs were performed to compare (1) the HOA subpopulations with the HC and (2) the THR subpopulations with the HC, both for the variables age, body height, body mass, BMI and walking speed. Two separate Chi-squared tests were performed to compare the distribution of sex within HOA subpopulations and within THR subpopulations. A Kruskal-Wallis H test was used to compare the KL score of the affected leg between HOA subpopulations. A two-way mixed model ANOVA was conducted with time (HOA vs. THR) as the within-subject factor and subpopulation as the between-subject factor to analyze changes in body mass, BMI and walking speed. Only patients who participated in both examinations were included in this repeated measure analysis.

For statistically significant ANOVA results, post-hoc tests (paired, or unpaired t-tests as appropriate) were conducted with Bonferroni correction for multiple comparisons. When data did not meet the assumptions of parametric testing, the following non-parametric tests were applied: Kruskal-Wallis H test (instead of one-way ANOVA), Scheirer-Ray-Hare test (instead of two-way repeated measures ANOVA), Wilcoxon signed-rank test (instead of paired t-tests) and Mann-Whitney U test (instead of unpaired t-tests).

Statistical parametric mapping with the spm1d package [40] was used to compare biomechanical waveforms. Two comparisons were performed: (1) between each identified HOA subpopulation as well as each THR subpopulation and HC (unpaired t-tests) and (2) between those participants represented in a subpopulation before and after total hip replacement (HOA vs. THR, paired t-tests).

The change over time of the GOGS within the subpopulations (HOA vs. THR) was done with a paired t-test for each of the subpopulations. COGS distributions were visualized with violin plots using the seaborn package [41] in Python (version 3.12, Python Software Foundation, Wilmington, Delaware, United States).

For all statistical tests, a two-sided p-value of < 0.05 was considered statistically significant. Effect sizes were calculated as follows: Cohen’s d for t-tests, eta squared $\:{(\eta\:}_{G}^{2})$ for ANOVAs and rank-biserial correlation (r) for non-parametric tests. Unless otherwise stated, results were reported as means with corresponding 95% confidence intervals (95% CI).

## Results

### Identification of subpopulations with hip OA

**Fig. 2:**
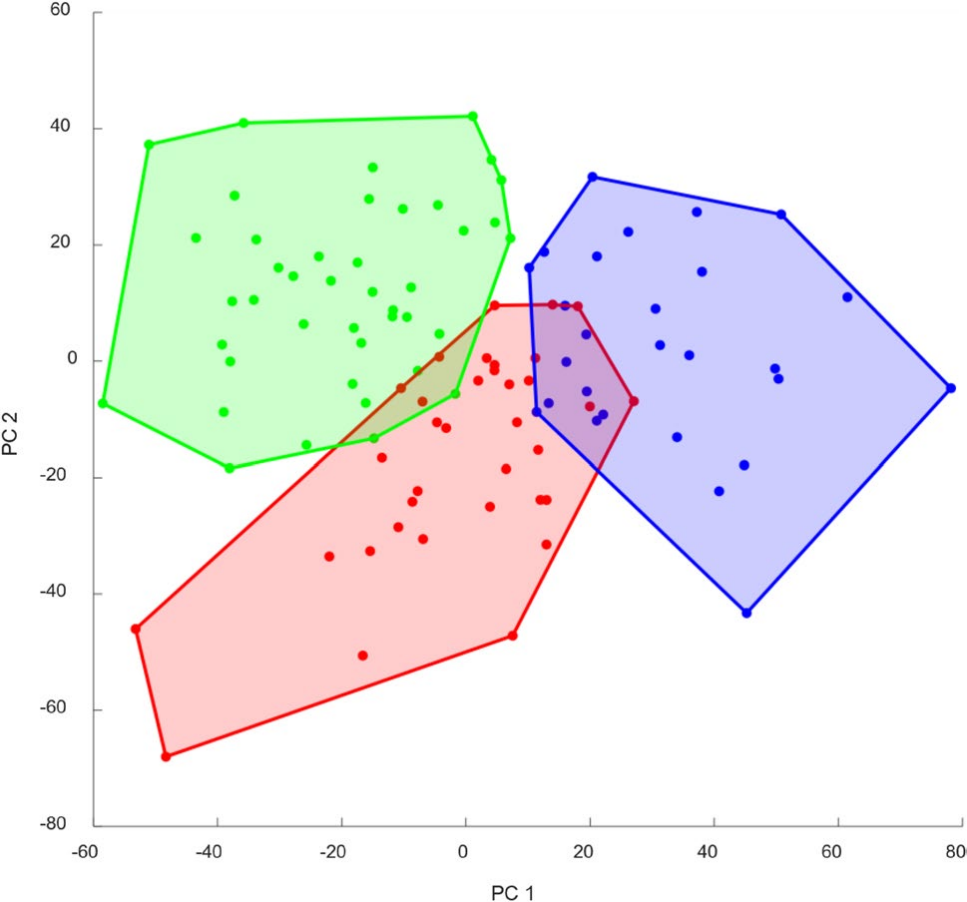
Two-dimensional visualization of the three identified subpopulations before total hip replacement based on the first two principal components (PC1 and PC2). Lines connect the outermost patients of each subpopulation to enhance visualization. Subpopulation with hip osteoarthritis HOA1 = blue, HOA2 = red, HOA3 = green

A total of 14 PCs were required to explain 90% of the variance across all biomechanical waveforms. Hierarchical clustering of the HOA dataset identified three distinct subpopulations, denoted as HOA1, HOA2, and HOA3. These subpopulations comprised of 27, 42, and 35 patients, respectively (Table 2). Five of the 109 HOA patients, two of whom were also included in the THR group, could not be consistently assigned to any single subpopulation and were therefore excluded from subsequent subpopulation-based analyses. A graphical representation of the three identified subpopulations is provided in Figure 2.

**Table 2:** Descriptive statistics for all subpopulations with the results of the one-way ANOVAs and the distribution tests

Variable	HOA1	HOA2	HOA3	THR1	THR2	THR3	HC	ANOVA (HOA vs. HC)	ANOVA (THR vs. HC)
N	27	42	35	16	27	18	56	/	/
Age [years]	64.9 [61.5 68.3]	61.8 [58.6 65.0]	57.8 [53.8 61.8]	64.2 [59.5 68.9]	62.7 [58.6 66.9]	60.2 [55.3 65.2]	63.5 [61.5 65.5]	F(3, 156) = 3.58; *p* = 0.015*;$\:{{\upeta\:}}_{\text{G}}^{2}\:$= 0.06	F(3, 113) = 0.75; *p* = 0.524;$\:{{\upeta\:}}_{\text{G}}^{2}\:$= 0.02
Body height [m]	1.71 [1.68 1.75]	1.68 [1.65 1.70]	1.73 [1.70 1.76]	1.74 [1.70 1.79]	1.70 [1.67 1.72]	1.73 [1.70 1.76]	1.68 [1.66 1.71]	F(3, 156) = 3.25; *p* = 0.023*;$\:{{\upeta\:}}_{\text{G}}^{2}\:$= 0.06	F(3, 113) = 3.33; *p* = 0.022*;$\:{{\upeta\:}}_{\text{G}}^{2}$ = 0.08
Body mass[kg]	84.1 [78.2 89.9]	76.7 [72.1 81.3]	82.3 [77.6 87.0]	90.3 [83.1 97.5]	79.0 [73.4 84.7]	80.2 [74.9 85.5]	69.0 [65.8 72.2]	F(3, 156) = 10.68; *p* < 0.001*;$\:{{\upeta\:}}_{\text{G}}^{2}$ = 0.17	F(3, 113) = 13.96; *p* < 0.001*;$\:{{\upeta\:}}_{\text{G}}^{2}$ = 0.27
BMI [kg/m^2^]	28.6 [26.9 30.2]	27.1 [25.8 28.5]	27.5 [26.1 28.9]	29.7 [27.6 31.8]	27.5 [25.6 29.3]	26.7 [25.2 28.3]	24.2 [23.5 25.0]	F(3, 156) = 10.62; *p* < 0.001*;$\:{{\upeta\:}}_{\text{G}}^{2}$ = 0.17	F(3, 113) = 13.96; *p* < 0.001*;$\:{{\upeta\:}}_{\text{G}}^{2}$ = 0.27
Walking speed [m/s]	0.98 [0.89 1.07]	1.03 [0.98 1.08]	1.03 [0.98 1.09]	1.12 [1.02 1.22]	1.16 [1.11 1.21]	1.23 [1.15 1.31]	1.32 [1.28 1.36]	F(3, 156) = 36.98; *p* < 0.001*;$\:{{\upeta\:}}_{\text{G}}^{2}$ = 0.42	F(3, 113) = 11.16; *p* < 0.001*;$\:{{\upeta\:}}_{\text{G}}^{2}$ = 0.23

	**HOA1**	**HOA2**	**HOA3**	**THR1**	**THR2**	**THR3**		**Distribution Test (HOA)**	**Distribution Test (THR)**
Sex [male/female]	15/12	15/27	24/11	11/5	10/17	13/5		**χ**^**2**^(2) = 8.45; *p* = 0.015*	**χ**^**2**^(2) = 6.91; *p* = 0.032*

Values are mean and 95% Confidence Interval for all variables

*HOA* Hip Osteoarthritis, *THR* Total Hip Replacement, *HC* Healthy Controls, *N* Number of individuals, *BMI* Body Mass Index, *F* Test-value for the parametric tests, Generalized eta squared, *χ*^*2*^ Chi-squared test; Level of significance ≤ 0.05; *marks a significant result

### Patient characteristics of subpopulations with hip OA

The patient characteristics of the three HOA subpopulations are presented in Table 2. The one-way ANOVA showed a significant main effect for all patient characteristics. Detailed pairwise comparisons are provided in the Supplementary Material (S.Table 1) and indicated that the subpopulation HOA1 was significantly older compared to HOA3 (64.9 [61.5, 68.3] years vs. 57.8 [53.8, 61.8] years, *p* = 0.023). HOA3 demonstrated a greater body height than HOA2 (1.73 [1.70, 1.79] m vs. 1.68 [1.65, 1.70] m, *p* = 0.021). Sex distributions differed within HOA subpopulations (**χ**^**2**^(2) = 8.45; *p* = 0.015): HOA1, 15 male/12 female patients; HOA2, 15 male/27 female patients; HOA3, 24 male/11 female patients. The KL score of the affected leg differed within HOA subpopulations (H(2, 162) = 12.68; *p* = 0.002; $\:{{\upeta\:}}_{\text{G}}^{2}$ = 0.11). Pairwise comparisons showed that HOA2 had a lower KL score (median = 3.0 [Interquartile range (IQR): 3–3]) compared with HOA1(3.5 [IQR: 3–4], *p* = 0.002) and HOA3 (3.4 [IQR: 3–4], *p* = 0.003, S.Table 2). When compared to HC, HOA3 was younger (*p* = 0.038) and taller (*p* = 0.031). All HOA subpopulations demonstrated greater body mass (p < 0.020), BMI (*p *< 0.001) and lower walking speeds (*p* < 0.001) than HC (S.Table 1).

### Biomechanical characterization of subpopulations with hip OA

The classification results are presented in Table 3. Individual classification rates for the three HOA subpopulations ranged from 63.7% to 90.4%. The proportion of patients classified as pathologic before total hip replacement varied between 51.4% and 85.2% for the HOA subpopulations and decreased to 27.8% to 51.8% after total hip replacement. For the HOA subpopulations, sensitivity ranged from 0.486 to 0.815, specificity from 0.750 to 0.929, and ROC-AUC from 0.689 to 0.919. Following total hip replacement, sensitivity decreased to 0.278 to 0.519, whereas specificity increased to 0.893 to 0.947, with corresponding ROC-AUC values between 0.568 and 0.913. The five most influential biomechanical waveforms, derived from the SVM-specific Shapley values, are listed in Table 4.

**Table 3:** Classification results for the three subpopulations at the two examinations

Subgroup	Classification rate [%]	Classified as HOA [*N*]	Classified as HC [*N*]	Pathologic ratio [%]	Sensitivity	Specificity	ROC-AUC
HOA1	90.4	23	4	85.2%	0.815	0.929	0.919
HOA2	73.5	26	16	61.9%	0.595	0.839	0.777
HOA3	63.7	18	17	51.4%	0.486	0.75	0.689
THR1	/	7	9	43.8%	0.438	0.947	0.913
THR2	/	14	13	51.9%	0.519	0.893	0.737
THR3	/	5	13	27.8%	0.278	0.929	0.568

*HOA *Hip Osteoarthritis,* THR *Total Hip Replacement,* HC *Healthy Controls,* N *Number of individuals,* ROC-AUC *Area Under the Receiver Operating Characteristic Curve

**Table 4:** The five most important biomechanical waveforms for the three SVM that classify an individual as patient with hip osteoarthritis. The number of the SVM corresponds to the HOA subpopulation.

Importance	SVM 1 (0.05–9.57)	SVM 2 (0.15–3.62)	SVM 3 (0.19–3.83)
1	Hip rotation angle (9.57)	Hip flexion moment (3.62)	Hip rotation angle (3.83)
2	Hip flexion moment (8.43)	Knee flexion angle (3.22)	Hip abduction angle (3.13)
3	Knee flexion angle (7.96)	Hip rotation angle (3.04)	Hip flexion angle (2.76)
4	Hip flexion angle (6.17)	Hip flexion angle (2.27)	Knee rotation angle (2.76)
5	Hip rotation moment (4.52)	Hip rotation moment (1.62)	Pelvic tilt (2.62)

The numbers in brackets represent the range of the importance of all biomechanical waveforms in the subpopulation-specific SVM

*SVM* Support Vector Machine

**Fig. 3:**
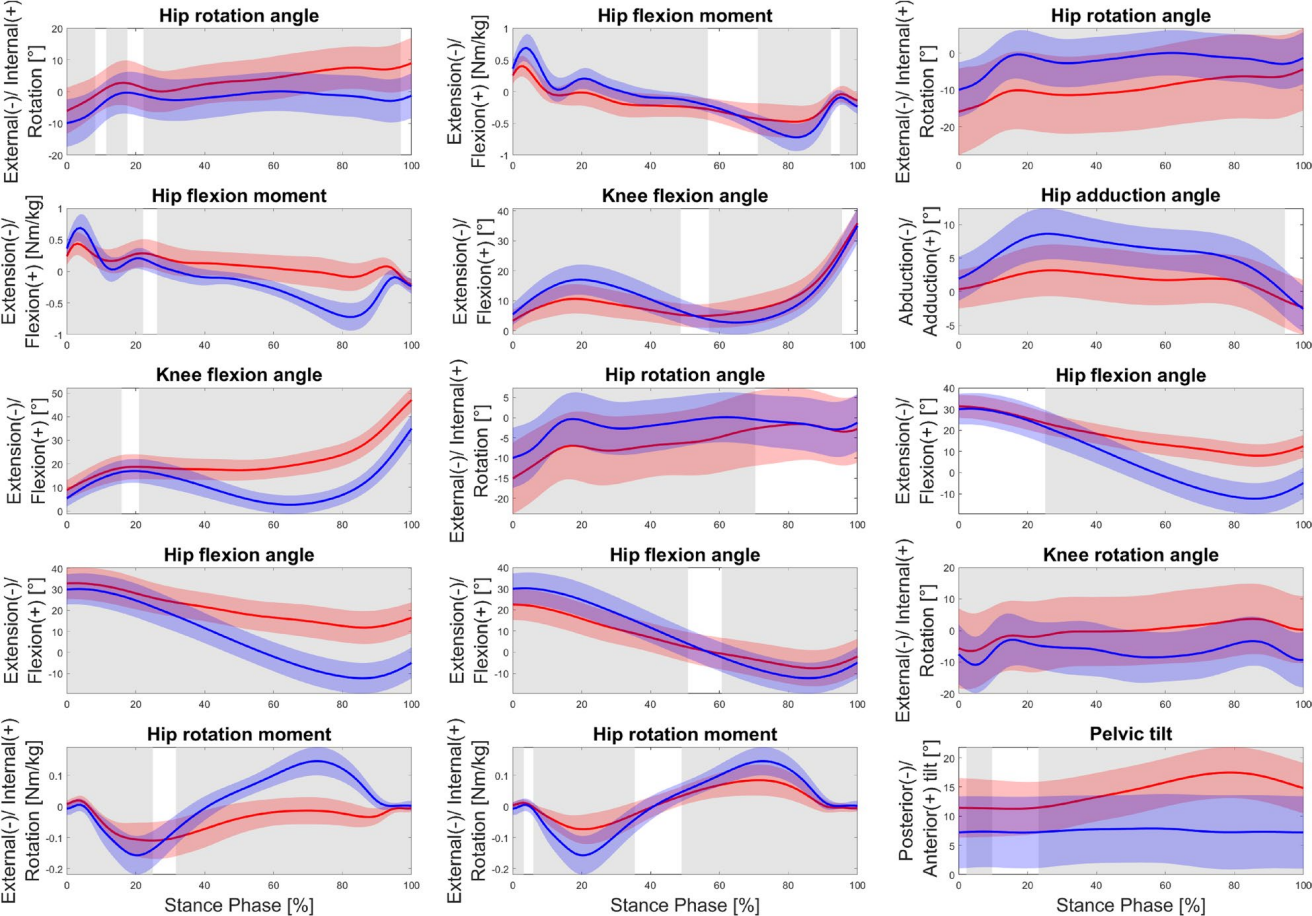
Differences between the five most important biomechanical waveforms for the three subpopulations before total hip replacement (red; HOA1 (left), HOA2 (middle), HOA3 (right)) and healthy controls (blue; HC). Level of significance < 0.05; The grey-shaded areas indicate significant differences

Figure 3 illustrates the time-dependent differences from HC for these five key biomechanical waveforms of the three subpopulations. Differences between HOA subpopulations and HC were observed over 74.9% to 100.0% of the stance phase. HOA1 showed greater internal hip rotation, higher hip and knee flexion, and lower sagittal and transversal hip moment ranges compared with HC. HOA2 demonstrated higher external hip rotation, lower hip flexion during early stance, lower hip extension during late stance, lower knee flexion in early stance, higher knee flexion in late stance, and lower hip sagittal and transversal moment ranges relative to HC. HOA3 showed higher external hip rotation, reduced hip adduction, higher hip flexion, reduced external knee rotation, and increased pelvis anterior tilt compared with HC.

### Patient characteristics of subpopulations after total hip replacement

The patient characteristics for the three subpopulations after total hip replacement (THR1, THR2, THR3) are summarized in Table 2, with detailed post-hoc comparisons provided in the Supplementary Materials (S.Table 3). THR1 patients were heavier than those in THR2 (90.3 [83.1, 97.5] kg vs 79.0 [73.4, 84.7] kg, p = 0.041). Sex distributions differed within THR subpopulations (χ2(2) = 6.91; p = 0.032): THR1, 11 male/5 female; THR2, 10 male/17 female; THR3, 13 male/5 female. When compared to HC, all subpopulations still had greater body mass (p < 0.008) and BMI (p < 0.002). THR3 reached the greatest improvement in walking speed reaching 1.23 [1.15, 1.31] m/s), which was only slightly below the value of HC (1.32 [1.28, 1.36] m/s, p = 0.120). THR1 and THR2 still had lower walking speeds (*p* < 0.002).

When comparing pre- and post-surgical subpopulations (HOA vs. THR) (Table 5), THR2 showed increased body mass and BMI (p = 0.002) relative to HOA2. All subpopulations showed significant improvements in walking speed after total hip replacement compared with their HOA conditions (p < 0.003, S.Table 4).

**Table 5:** Descriptive statistics of the subpopulations with only the patients who were included in the pre- and post- surgery dataset as well as the results for the two-way mixed model ANOVA (factors: subpopulation and time of measurement)

Variable	HOA1	HOA2	HOA3	THR1	THR2	THR3	ANOVA (SUB)	ANOVA (Time)	ANOVA (Interaction)
N	16	27	18	16	27	18			
Age [years]	63.1 [58.4 67.8]	61.7 [57.6 65.8]	59.0 [54.2 63.8]	64.2 [59.5 68.9]	62.7 [58.6 66.9]	60.2 [55.3 65.2]	F(2, 101) = 3.642); *p* = 0.030*;$\:{{\upeta\:}}_{\text{G}}^{2}$ = 0.07		
Body height [m]	1.74 [1.70 1.77]	1.70 [1.67 1.72]	1.73 [1.70 1.76]	1.74 [1.70 1.79]	1.70 [1.67 1.72]	1.73 [1.70 1.76]	F(2, 101) = 3.86); *p* = 0.024*;$\:{{\upeta\:}}_{\text{G}}^{2}$ = 0.07		
Body mass [kg]	89.23 [82.3 96.1]	77.6 [72.3 83.0]	79.6 [74.9 84.3]	90.3 [83.1 97.5]	79.0 [73.4 84.7]	80.2 [74.9 85.5]	F(2, 58) = 4.39); *p* = 0.017*;$\:{{\upeta\:}}_{\text{G}}^{2}$ = 0.13	F(1, 58) = 10.01); *p* = 0.002*;$\:{{\upeta\:}}_{\text{G}}^{2}$ = 0.00	F(2, 58) = 0.51); *p* = 0.602;$\:{{\upeta\:}}_{\text{G}}^{2}$ = 0.00
BMI [kg/m^2^]	29.4 [27.4 31.4]	27.0 [25.3 28.7]	26.6 [25.2 27.0]	29.7 [27.6 31.8]	27.5 [25.6 29.3]	26.7 [25.2 28.3]	F(2, 58) = 2.62); *p* = 0.082;$\:{{\upeta\:}}_{\text{G}}^{2}$ = 0.08	F(1, 58) = 7.19); *p* = 0.010*;$\:{{\upeta\:}}_{\text{G}}^{2}$ = 0.00	F(2, 58) = 0.52); *p* = 0.599;$\:{{\upeta\:}}_{\text{G}}^{2}$ = 0.00
Walking speed [m/s]	0.96 [0.84 1.09]	1.05 [0.99 1.12]	1.04 [0.96 1.11]	1.12 [1.02 1.22]	1.16 [1.11 1.21]	1.23 [1.15 1.31]	F(2, 58) = 1.69); *p* = 0.193;$\:{{\upeta\:}}_{\text{G}}^{2}$ = 0.05	F(1, 58) = 61.47); *p* < 0.001*;$\:{{\upeta\:}}_{\text{G}}^{2}$ = 0.17	F(2, 58) = 2.15); *p* = 0.125;$\:{{\upeta\:}}_{\text{G}}^{2}$ = 0.01

Values are mean and 95% Confidence Interval for all variables apart from Kellgren-Lawrence score, which is reported as mean and interquartile range

*HOA *Hip Osteoarthritis, *THR* Total Hip Replacement, *N* Number of individuals, *KL* Kellgren-Lawrence score, *BMI* Body Mass Index, *SUB* Between subpopulation effect, Time = within subpopulation effect; F = test-value for the parametric tests;$\:{{\upeta\:}}_{\text{G}}^{2}$= generalized eta squared, Level of significance ≤ 0.05; * marks a significant result

### Biomechanical characteristics and COGS of subpopulations after total hip replacement

**Fig. 4:**
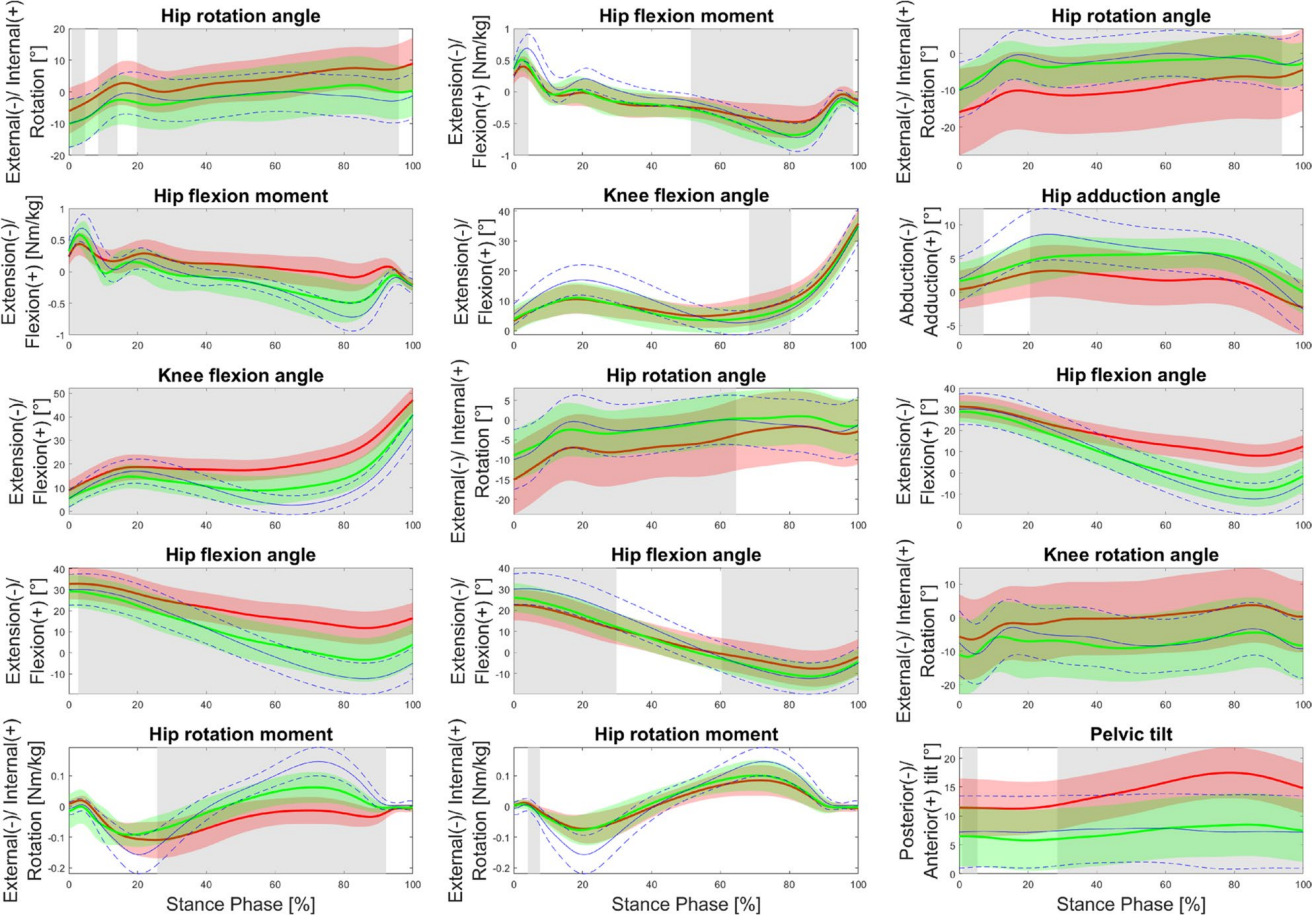
Differences between the five most important biomechanical waveforms for the three subpopulations before (red; HOA1 (left), HOA2 (middle), HOA3 (right)) and after total hip replacement (green; THR1 (left), THR2 (middle), THR3 (right)). Level of significance ≤ 0.05; The grey-shaded areas indicate significant differences. The blue lines represent the mean and standard deviation of the healthy controls (HC)

Figure 4 depicts the time-dependent differences in the five most influential biomechanical waveforms between HOA and THR subpopulations. Across all subpopulations, the waveforms demonstrated differences from the HOA patterns, with changes trending towards the HC patterns. For all biomechanical waveforms, THR1 and THR3 exhibited pronounced changes compared to HOA, showing differences over 66.6 to 100% of the stance phase. In contrast, THR2 showed fewer changes, with the most notable differences occurring for the hip rotation angle (over 69.5% of the stance phase), whereas knee flexion angle and hip rotation moment differed only over short periods of the stance phase (12.2% and 3.5%, respectively).

**Fig. 5:**
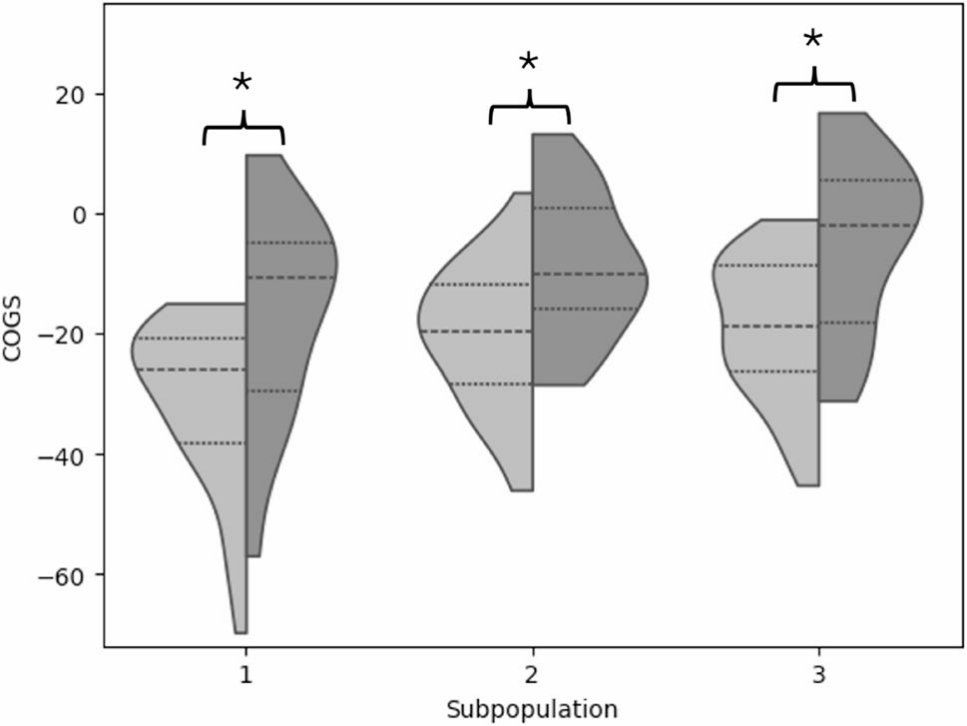
Violin-plot for the classifier-oriented gait score (GOGS) quantifying gait function. The left side (light grey; HOA1, HOA2, HOA3) of each violin are the subpopulation-specific COGS before and on the right side (dark grey; THR1, THR2, THR3) are the subpopulation-specific COGS after total hip replacement. Level of significance ≤ 0.05; * marks a significant result

For all subpopulations, COGS increased from HOA to THR (HOA1 vs THR1: t(15) = 5.22, *p* < 0.001; HOA2 vs THR2: t(26) = 5.37, *p* < 0.001; HOA3 vs THR3: t(17) = 5.39, *p* < 0.001; Figure 5), indicating an overall improvement in gait quality following surgery. 

## Discussion

This study aimed to identify and characterize subpopulations of patients with hip OA based on their distinct adaptations in gait kinematics and joint moments, and to evaluate how these subpopulations respond to total hip replacement. The findings revealed three biomechanically distinct subpopulations, each characterized by unique gait biomechanics, patient characteristics, and responses to total hip replacement. These results confirm our hypotheses and underscore the heterogeneity of hip OA, emphasizing the need for more personalized clinical management

### Differences among subpopulations with hip OA

Our data-driven approach identified three demographically and biomechanically distinct hip OA subpopulations that differed in age, sex, radiographic disease severity, body composition, and walking speed; all factors known to influence both functional impairment and surgical outcomes [42]. Specifically, HOA1 was the oldest subpopulation and had the highest KL score, whereas HOA3 was the youngest, tallest, and had a lower KL score than HOA1, but higher than HOA2. All subpopulations walked slower and had higher body mass and BMI compared with HC. The identification of these subpopulations through data-driven clustering is plausible, as patient characteristics age, body composition, and walking speed are known to influence gait biomechanics [43], which served as the basis for clustering. These insights emphasize that patients should not be categorized solely by radiographic severity, as demographic and functional characteristics can provide additional insight into disease manifestation and risk stratification [44–46]. Age, body composition and walking speed, often underemphasized in routine clinical evaluation, could enhance the identification of high-risk individuals and guide more individual treatment strategies as well as reduce unnecessary interventions [47, 48]. Interestingly, the clustering process, which did not include anthropometrics or sex information, revealed one predominantly female (HOA2) and one predominantly male (HOA3) subpopulation. This aligns with previous studies [20, 49] reporting sex-specific gait patterns in OA patients, suggesting that our workflow effectively captures biomechanical-relevant features.

 Regarding the classification based on gait biomechanics, HOA1 showed the highest separability from HC (classification rate of 90.4%), followed by HOA2 (73.5%) and HOA3 (63.7%), indicating distinct degrees of deviations in gait biomechanics. These rates were comparable or slightly lower than previously reported [18, 20] likely reflecting milder gait alterations in HOA2 and HOA3. The lower rates of these groups supports the idea that their biomechanical changes from HC are less pronounced [21]. This is further supported by the reduced sensitivity (true-positive rate) in the THR subpopulations, meaning fewer participants are classified as pathological.

 Of the 15 most influential biomechanical waveforms identified (five per subpopulation), 11 originated from the hip, three from the knee and one from the pelvis. This distribution reflects the central role of hip mechanics in gait pathology. From a clinical perspective, the subpopulation-specific adaptations observed here may explain the variability in previous literature on compensatory strategies in hip kinematics and kinetics [10]. Interestingly, both internal and external hip rotations were observed: HOA1 displayed a flexed and internally rotated hip; HOA2 showed and extended and externally rotated hip; and HOA3 demonstrated a flexed and externally rotated hip. These findings mirror the variability found in an earlier meta-analysis regarding hip rotation in hip OA [8] and provide a biomechanical rationale for conflicting results. Notably, HOA3, comprising younger, taller patients with moderate radiographic findings, demonstrated distinct gait adaptations including reduced hip adduction and increased anterior pelvic tilt during late stance [13, 50]. These biomechanical gait adaptations may reflect compensatory mechanisms in younger, more active individuals and highlight the need for earlier or targeted interventions in this subpopulation to delay disease progression or optimize surgical timing [51, 52].

### Subpopulation-specific functional improvement after total hip replacement

 All subpopulations demonstrated functional improvement following total hip replacement, particularly in walking speed, with THR3 approaching the level of healthy controls. Walking speed is a robust surrogate for overall mobility and quality of life [12], underscoring the effectiveness of total hip replacement in restoring function across diverse subpopulations [53, 54]. The observed post-surgery weight gain in THR2 warrants attention. While often considered undesirable, such an increase may indicate improved physical capacity or increased muscle mass, both of which can enhance function [53, 55]. Nevertheless, this underscores the importance of incorporating weight management and strength training into postoperative rehabilitation protocols.

Reductions in gait compensations, as quantified by the COGS, further support the efficacy of total hip replacement in restoring biomechanical function. Clinically, metrics like the COGS could enable standardized, automated gait monitoring of rehabilitation progress and facilitate data-driven treatment evaluation [18].

 Biomechanical waveform analysis revealed overall movement patterns shifting towards HC, consistent with prior meta-analysis [10]. Interestingly, THR1, which was initially the most impaired subpopulation, exhibited the greatest biomechanical normalization, suggesting that patients with more pronounced preoperative deficits may derive larger relative benefits from surgery [42]. In contrast, THR2, which presented milder impairments, showed smaller postoperative changes, highlighting a possible ceiling effect in patients with milder preoperative impairments [42].

 Together, the results highlight the subpopulation-dependent nature of surgical recovery following total hip replacement and support the clinical importance of tailoring rehabilitation strategies to individual biomechanical adaptations to maximize functional gains.

### Implications for personalized hip OA therapy

 The identification of subpopulation-specific adaptations in gait kinematics and joint moments highlights opportunities for advancing personalized treatment and rehabilitation strategies in hip OA. Standardized treatment protocols may not sufficiently address the heterogeneous functional limitations and compensatory mechanisms across patient subpopulations. For example, individuals in HOA1 may benefit from interventions targeting hip extension and reducing compensatory knee stiffness, while HOA2 may require rehabilitation strategies to improve hip rotation control. Comprehensive biomechanical assessment before and after surgery could enable clinicians to tailor interventions more precisely, optimizing both short-term and long-term recovery [10, 56]. Moreover, the persistence of certain gait compensations after total hip replacement underscores the need to move beyond generic mobility training toward targeted biomechanical retraining [57, 58]. In a realistic clinical setting, a potential workflow may involve automatic classification of new patients with hip OA via their gait analysis: gait data are automatically processed (normalization and PCA) and assigned to a subpopulation using the clustering model (e.g. HOA2). Based on this classification, the COGS is calculated using the corresponding SVM, and an estimation of likely functional improvement after surgery can be made (e.g., moderate improvement for HOA2 with certain complications). This prediction can be used to suggest an individualized rehabilitation protocol, for instance rotational control training and weight management for a patient in HOA2. In this context, mobile measurement technologies could further facilitate the collection of detailed biomechanical data to advance the individual approach [59], while integration with adjacent predictive systems (e.g. radiological forecasting models [7, 60] or predicting postoperative complications [6, 61]) ultimately enhances the development of more personalized therapeutic pathways [19].

### Limitations

 Several limitations should be considered when interpreting the results. First, sex differences within the subpopulations were not explicitly analyzed due to limited subgroup sizes and imbalanced sex distribution. Although the subpopulations containing mainly male or female patients showed consistent post-surgical changes, future studies with larger and more balanced cohorts and a more specific sex analysis might yield different results [20, 49, 62]. Second, only the affected limb was analyzed. While this approach ensured that clustering reflected adaptations specific to the pathological joint, it precluded analysis of contralateral compensations, which are known to occur in hip OA and after surgery [11, 63]. Including both legs in future studies (effectively doubling the dataset) would allow characterization of subpopulation‑specific contralateral compensatory patterns, though care must be taken to avoid diluting pathology specific effects. Here, subpopulations driven by the contralateral leg could be created, potentially misleading therapeutic implications. Third, surgical factors such as the operation surgeon, approach, and implant type could influence surgical outcomes [64–67]. In the present study data were collected over a 10- year period with multiple surgeons involved and implant types used. However, post hoc chi-squared tests revealed no uneven distribution of these factors across the subpopulations (p >0.400). Nonetheless, standardized surgery studies (one surgeon and same implant) could minimize these sources of variability. Another limitation concerns how well each patient fits into their subpopulation. The silhouette values obtained from k-means clustering were moderate, typically above 0.25, suggesting a systematic but potentially limited fit [37]. Finally, although nonlinear dimensionality-reduction methods or hyperparameter tuning could improve ML model accuracy, such approaches might compromise clinical interpretability, which is essential for translational applications in healthcare.

## Conclusion

 This study identified clinically meaningful heterogeneity among patients with hip OA, underscoring the potential of gait biomechanics to inform individualized treatment decisions and guide postoperative rehabilitation. The observed subpopulation-specific differences in gait adaptations and surgical responses highlight the potential of ML-based classification to support a more personalized approach to hip OA management. Integrating biomechanical gait data and explainable ML into clinical practice could enhance outcome prediction, enable tailored interventions, and ultimately improve quality of life for patients undergoing total hip replacement.

## Supplementary Information

Supplementary Material 1

## Data Availability

The datasets analyzed during the current study are not publicly available due to patient privacy restrictions but are available from the corresponding author on reasonable request.
