# Supplementary material for: Explainable machine learning for orthopedic decision-making: predicting functional outcomes of total hip replacement from gait biomechanics
Source: Arthritis Res Ther. 2025 Dec 23;27:229. doi: 10.1186/s13075-025-03709-2 (PMC12743397; doi:10.1186/s13075-025-03709-2)
Supplement: Supplementary file 1 — Supplementary Material 1 [file 13075_2025_3709_MOESM1_ESM.docx]

# Supplementary material

S.Table 1. Results of the pairwise comparisons between the HOA subpopulations and healthy controls for the patient characteristics variables.

| **Variable** | **Comparison** | **t-statistic** | **DF** | **p-value** | **Means** | **Effect size d** | **95% confidence**  **intervals** |
| --- | --- | --- | --- | --- | --- | --- | --- |
| Age [years] | HOA1, HC | 1.49 | 51.68 | 1.000 | 64.89, 63.39 | 0.17 | [-5.33 2.56] |
|  | HOA2, HC | 2.75 | 59.97 | 1.000 | 61.81, 63.39 | 0.19 | [-2.08 10.13] |
|  | HOA3, HC | 1.59 | 69.02 | 0.038* | 57.80, 63.39 | 0.58 | [1.28 10.10] |
|  | HOA1, HOA2 | 1.33 | 62.21 | 0.564 | 64.89, 61.8 | 0.32 | [-1.55 7.70] |
|  | HOA1, HOA3 | 2.75 | 59.97 | 0.023* | 64.89, 57.8 | 0.69 | [1.94 12.20] |
|  | HOA2, HOA3 | 1.59 | 69.02 | 0.351 | 61.81, 57.8 | 0.37 | [-1.03 9.05] |
| Body height [m] | HOA1, HC | 1.49 | 51.68 | 0.429 | 1.71, 1.68 | 0.44 | [-0.07 0.01] |
|  | HOA2, HC | -0.26 | 91.55 | 1.000 | 1.68, 1.68 | 0.05 | [-0.03 0.04] |
|  | HOA3, HC | 2.63 | 78.95 | 0.031* | 1.73, 1.68 | 0.56 | [-0.08 -0.01] |
|  | HOA1, HOA2 | 1.66 | 52.87 | 0.309 | 1.71, 1.68 | 0.41 | [-0.007 0.07] |
|  | HOA1, HOA3 | -0.74 | 52.41 | 1.000 | 1.71, 1.73 | 0.19 | [-0.06 0.03] |
|  | HOA2, HOA3 | -2.78 | 73.7 | 0.021* | 1.68, 1.73 | 0.64 | [-0.09 -0.02] |
| Body mass [kg] | HOA1, HC | 4.62 | 42.87 | <0.001* | 84.06, 68.99 | 1.12 | [-21.60 -8.49] |
|  | HOA2, HC | 2.79 | 77.38 | 0.020* | 76.72, 68.99 | 0.58 | [-13.30 -2.21] |
|  | HOA3, HC | 4.76 | 64.91 | <0.001* | 82.34, 68.99 | 1.04 | [-19.00 -7.74] |
|  | HOA1, HOA2 | 2.02 | 55.38 | 0.146 | 84.06, 76.72 | 0.50 | [0.05 14.63] |
|  | HOA1, HOA3 | 0.47 | 53.75 | 1.000 | 84.06, 82.34 | 0.12 | [-5.63 9.07] |
|  | HOA2, HOA3 | -1.73 | 74.06 | 0.261 | 76.72, 82.34 | 0.40 | [-12.07 0.84] |

S.Table 1. Continued.

| **Variable** | **Comparison** | **t-statistic** | **DF** | **p-value** | **Means** | **Effect size d** | **95% confidence**  **intervals** |
| --- | --- | --- | --- | --- | --- | --- | --- |
| BMI [kg/m^2^] | HOA1, HC | 4.88 | 37.51 | <0.001* | 28.55, 24.24 | 1.22 | [-6.11 -2.52] |
|  | HOA2, HC | 3.83 | 66.24 | <0.001* | 27.14, 24.24 | 0.80 | [-4.41 -1.39] |
|  | HOA3, HC | 4.10 | 53.58 | <0.001* | 27.47, 24.24 | 0.92 | [-4.81 -1.65] |
|  | HOA1, HOA2 | 1.36 | 56.54 | 0.534 | 28.55, 27.14 | 0.34 | [-0.66 3.50] |
|  | HOA1, HOA3 | 1.02 | 55.61 | 0.933 | 28.55, 27.47 | 0.26 | [-1.04 3.21] |
|  | HOA2, HOA3 | -0.09 | 73.44 | 1.000 | 27.14, 27.47 | 0.08 | [-2.24 1.58] |
| Walking speed [m/s] | HOA1, HC | 7.18 | 38.6 | <0.001* | 0.98,1.32 | 1.78 | [0.24 0.44] |
|  | HOA2, HC | 8.69 | 83.5 | <0.001* | 1.03, 1.32 | 1.79 | [0.22 0.36] |
|  | HOA3, HC | 8.14 | 67.3 | <0.001* | 1.03, 1.32 | 1.77 | [0.22 0.36] |
|  | HOA1, HOA2 | -1.00 | 45.34 | 0.969 | 0.98, 1.03 | 0.25 | [-0.15 0.05] |
|  | HOA1, HOA3 | -1.04 | 47.31 | 0.906 | 0.98, 1.03 | 0.27 | [-0.16 0.05] |
|  | HOA2, HOA3 | -0.09 | 72.69 | 1.000 | 1.03, 1.03 | 0.02 | [-0.08 0.07] |

Level of significance ≤ 0.05; * marks a significant result. Abbreviations: HOA1-3 = subpopulation with Hip Osteoarthritis; HC = healthy controls; BMI = Body Mass Index.

S.Table 2. Results of the pairwise, non-parametric comparisons (Mann-Whitney-U-Test) between the subpopulations for the KL score.

| **Variable** | **Comparison** | **z-value** | N | **Means** | **p-value** | **Effect size r** | **95% confidence**  **intervals** |
| --- | --- | --- | --- | --- | --- | --- | --- |
| KL of the ipsilateral leg | HOA1, HOA2 | -3.16 | 27, 42 | 3.48, 3.00 | 0.002* | 0.38 | [0.0005 1.00] |
|  | HOA1, HOA3 | -0.08 | 27, 35 | 3.48, 3.43 | 0.936 | 0.01 | [-0.0005 0.0003] |
|  | HOA2, HOA3 | -2.93 | 42, 35 | 3.00, 3.43 | 0.003* | 0.33 | [-1.00 -0.0005] |

Level of significance ≤ 0.05; * marks a significant result. Abbreviations: KL = Kellgren-Lawrence score; HOA1-3 = subpopulation with Hip Osteoarthritis.

S.Table 3. Results of the pairwise comparisons between the THR subpopulations and healthy controls for the patient characteristics variables.

| **Variable** | **Comparison** | **t-statistic** | **DF** | **p-value** | **Means** | **Effect size d** | **95% confidence**  **intervals** |
| --- | --- | --- | --- | --- | --- | --- | --- |
| Body mass [kg] | THR1, HC | 5.73 | 22.21 | <0.001* | 90.29, 68.99 | 1.68 | [-29.00 -13.60] |
|  | THR2, HC | 3.17 | 44.14 | 0.008* | 79.03, 68.99 | 0.76 | [-16.40 -3.65] |
|  | THR3, HC | 3.75 | 31.61 | 0.002* | 80.19, 68.99 | 0.99 | [-17.30 -5.11] |
|  | THR1, THR2 | 2.60 | 33.17 | 0.041* | 90.29, 79.03 | 0.81 | [2.45 20.10] |
|  | THR1, THR3 | 2.40 | 28.71 | 0.069 | 90.29, 80.19 | 0.83 | [1.50 20.09] |
|  | THR2, THR3 | 0.31 | 42.63 | 1.000 | 79.03, 80.19 | <0.01 | [-8.69 6.37] |
| BMI [kg/m^2^] | THR1, HC | 5.25 | 19.61 | 0.001* | 29.69, 24.24 | 1.62 | [-7.62 -3.28] |
|  | THR2, HC | 3.33 | 35.27 | 0.006* | 27.47, 24.24 | 0.84 | [-5.19 -1.26] |
|  | THR3, HC | 3.00 | 25.92 | 0.018* | 26.75, 24.24 | 0.84 | [-4.23 -0.79] |
|  | THR1, THR2 | 1.69 | 36.21 | 0.300 | 29.69, 27.47 | 0.52 | [-0.45 4.90] |
|  | THR1, THR3 | 2.40 | 29.08 | 0.069 | 29.69, 26.75 | 0.83 | [0.44 5.45] |
|  | THR2, THR3 | 0.62 | 42.95 | 1.000 | 27.47, 26.75 | 0.18 | [-1.63 3.07] |
| Walking speed [m/s] | THR1, HC | 3.98 | 21.19 | 0.002* | 1.12, 1.20 | 0.08 | [0.10 0.30] |
|  | THR2, HC | 5.13 | 62.59 | <0.001* | 1.16, 1.32 | 0.23 | [0.10 0.22] |
|  | THR3, HC | 2.15 | 28.48 | 0.120 | 1.23, 1.32 | 0.70 | [0.004 -0.18] |
|  | THR1, THR2 | -0.78 | 22.99 | 1.000 | 1.12, 1.16 | 0.26 | [-0.15 0.07] |
|  | THR1, THR3 | -1.89 | 29.40 | 0.206 | 1.12, 1.23 | 0.65 | [-0.23 0.009] |
|  | THR2, THR3 | -1.63 | 30.82 | 0.342 | 1.16, 1.23 | 0.51 | [-0.16 0.018] |

Level of significance ≤ 0.05; * marks a significant result. Abbreviations: THR1-3 = subpopulation after Total Hip Replacement; HC = healthy controls; BMI = Body Mass Index.

S.Table 4. Results of the relevant pairwise comparisons for the factor time of measurement of the two-way repeated measures ANOVA

for the patient characteristics variables.

| **Variable** | **Comparison** | **t-statistic** | **DF** | **p-value** | **Means** | **Effect size r** | **95% confidence**  **intervals** |
| --- | --- | --- | --- | --- | --- | --- | --- |
| Body mass [kg] | HOA1, THR1 | -1.65 | 15 | 0.119 | 89.23, 90.29 | 0.41 | [-0.31 2.45] |
|  | HOA2, THR2 | -3.37 | 26 | 0.002* | 77.64, 79.03 | 0.65 | [0.54 2.22] |
|  | HOA3, THR3 | -0.94 | 17 | 0.360 | 79.57, 80.19 | 0.22 | [-0.77 2.02] |
| Walking speed [m/s] | HOA1, THR1 | -3.24 | 15 | 0.007* | 0.96, 1.12 | 0.78 | [0.05 0.26] |
|  | HOA2, THR2 | -3.99 | 26 | 0.001* | 1.05, 1.16 | 0.77 | [0.05 0.15] |
|  | HOA3, THR3 | -7.85 | 17 | *<*0.001* | 1.04, 1.23 | 1.85 | [0.14 0.24] |
| BMI [kg/m^2^] | HOA1, THR1 | -1.24 | 15 | 0.235 | 29.41, 29.69 | 0.31 | [-0.21 0.78] |
|  | HOA2, THR2 | -3.35 | 26 | 0.002* | 27.02, 27.47 | 0.64 | [0.17 0.72] |
|  | HOA3, THR3 | -0.75 | 17 | 0.462 | 26.56, 26.74 | 0.18 | [-0.34 0.71] |

Level of significance ≤ 0.05; * marks a significant result. Abbreviations: HOA1-3 = subpopulation with Hip Osteoarthritis; HC = healthy controls; THR1-3 = subpopulation after Total Hip Replacement; BMI = Body Mass Index.
